# **Effects of repetitive transcranial magnetic stimulation in children and young people with psychiatric disorders: a systematic review**

**DOI:** 10.1007/s00787-024-02475-x

**Published:** 2024-05-29

**Authors:** Lucy Gallop, Samuel J. Westwood, Amelia Hemmings, Yael Lewis, Iain C. Campbell, Ulrike Schmidt

**Affiliations:** 1 Centre for Research in Eating and Weight Disorders, Department of Psychological Medicine, Institute of Psychiatry, Psychology & Neuroscience, King’s College London, De Crespigny Park, PO Box 59, London, SE5 8AF UK; 2 Department of Psychology, School of Social Science, University of Westminster, London, W1W 6UW UK; 3 Institute of Psychiatry, Psychology and Neuroscience, King’s College London, London, SE5 8AB UK; 4 Hadarim Eating Disorder Unit, Shalvata Mental Health Centre, Hod Hasharon, Israel; 5 Sackler Faculty of Medicine, Tel-Aviv University, Tel-Aviv, Israel; 6 South London and Maudsley NHS Foundation Trust, London, UK

**Keywords:** rTMS, Children, Young people, Psychiatric disorders, Systematic review

## Abstract

Repetitive transcranial magnetic stimulation (rTMS) has demonstrated benefits in adults with psychiatric disorders, but its clinical utility in children and young people (CYP) is unclear. This PRISMA systematic review used published and ongoing studies to examine the effects of rTMS on disorder-specific symptoms, mood and neurocognition in CYP with psychiatric disorders. We searched Medline via PubMed, Embase, PsychINFO via OVID, and Clinicaltrials.gov up to July 2023. Eligible studies involved multiple-session (i.e., treatment) rTMS in CYP (≤ 25 years-old) with psychiatric disorders. Two independent raters assessed the eligibility of studies and extracted data using a custom-built form. Out of 78 eligible studies (participant *N* = 1389), the majority (k = 54; 69%) reported an improvement in at least one outcome measure of disorder-specific core symptoms. Some studies (k = 21) examined rTMS effects on mood or neurocognition,: findings were largely positive. Overall, rTMS was well-tolerated with minimal side-effects. Of 17 ongoing or recently completed studies, many are sham-controlled RCTs with better blinding techniques and a larger estimated participant enrolment. Findings provide encouraging evidence for rTMS-related improvements in disorder-specific symptoms in CYP with different psychiatric disorders. However, in terms of both mood (for conditions other than depression) and neurocognitive outcomes, evidence is limited. Importantly, rTMS is well-tolerated and safe. Ongoing studies appear to be of improved methodological quality; however, future studies should broaden outcome measures to more comprehensively assess the effects of rTMS and develop guidance on dosage (i.e., treatment regimens).

**Supplementary Information:**

The online version contains supplementary material available at 10.1007/s00787-024-02475-x.

## Introduction

Globally, mental health disorders affect 13–20% of children, adolescents, and young people (CYP) [1–3], although, evidence indicates that rates have increased during COVID-19 [4, 5]. Over 60% of all mental health disorders emerge before the age of 25 [6] which coincides with extensive age-related changes in brain organisation and function, making it a period of vulnerability and opportunity for early intervention of mental health issues in CYP [7]. Meta-analytic evidence of randomised controlled trials (RCTs) comparing psychosocial interventions with waiting list, or no intervention, generally show large effect sizes in CYP with psychiatric disorders (e.g [8]). . , , but little meta-analytic evidence exists for CYP who have not responded to first- and/or second-line treatments [9]. Moreover, psychotropic medications are widely prescribed off-label in CYP, and without a thorough risk-benefit analysis [10]. Overall, this highlights the need for novel, safe biotherapies as adjuncts or alternatives to currently available treatments [11].

Transcranial magnetic stimulation (TMS) is a non-invasive brain stimulation technique that uses an electromagnetic coil to stimulate neurones and modulate cortical excitability in a target brain region [12]. Repetitive TMS (rTMS) can induce neural effects that outlast the stimulation [13], with more durable changes occurring when rTMS is given in daily sessions over 1–6 weeks [14]. Depending on the stimulation frequency, rTMS can have facilitatory or inhibitory effects on cortical excitability. High-frequency rTMS (HF-rTMS; >5 Hz) generally increases cortical excitability and low-frequency rTMS (LF-rTMS; <1 Hz) generally decreases excitability [15, 16]. HF-rTMS and LF-rTMS remain classical protocols, but newer variants with shorter stimulation periods (49–190 s) also induce excitatory (intermittent theta burst stimulation; iTBS) or inhibitory (continuous theta burst stimulation; cTBS) cortical effects [17].

Although the precise mechanisms of action of neuromodulation procedures are unclear, long-lasting synaptic plasticity-related changes following rTMS are thought to emulate long-term potentiation/depression (LTP/LTD) [18]. Indeed, rTMS has demonstrated efficacy for improving symptoms in psychiatric disorders associated with cortical hyper- or hypo-excitability, and clinical guidelines recommend rTMS and/or iTBS as safe and effective treatments for major depressive disorder [19, 20] and obsessive-compulsive disorder [21] in adults.

In CYP, reviews suggest a comparable safety profile of rTMS to that in adults, with most adverse events being mild and overall, being uncommon [22, 23]. Other reviews have summarised the clinical effects of rTMS in CYP with treatment-resistant depression [24–26], neurodevelopmental disorders [27, 28], and in conditions other than depression [29] and these provide encouraging preliminary evidence. However, they are outdated and/or non-systematic, narrative reviews that focus exclusively on the effects of rTMS in a specific disorder, or on the safety and tolerability of rTMS in CYP, and, for example, do not examine the effects of rTMS on mood (in conditions other than depression) or on cognition. Accordingly, we have systematically reviewed studies investigating the effects of rTMS across psychiatric disorders in CYP to (1) evaluate the effects of rTMS on disorder-specific symptoms and impairments, (2) determine the effects of rTMS on mood and neurocognitive outcomes, (3) outline the populations and methodologies used in ongoing trials and unpublished data.

## Methods

We followed PRISMA 2020 (Preferred Reporting Items for Systematic Reviews and Meta-Analyses; [30]) guidelines.

### Protocol and registration

This study was pre-registered (see PROSPERO, ID: CRD42019158957; and [31]).

### Literature search

MEDLINE, EMBASE, and PsycINFO databases were searched using the following search terms: (repetitive transcranial magnetic stimulation or rTMS) AND (young people, child, adolescent, young adult, youth, boy, girl, paediatric, young people and young persons) AND (neuropsychiatric disorders, autism, ADHD, schizophrenia, mood disorder, bipolar, depression, anxiety, panic, OCD, Tourette’s, PTSD, acute stress disorder, substance abuse, eating disorders, personality disorder). The search was conducted on 28/01/21 and updated on 26/07/23. The reference lists of included studies were manually searched for additional relevant studies not identified by the database search. To identify ongoing/unpublished trials, we searched Clinicaltrials.gov, World Health Organisation International Clinical Trials Registry Platform (ICTRP) registry, the National Institute of Health (NIH) registry, the European Union Clinical Trials Register, and the International Standard Randomised Controlled Trials Number (ISRCTN) registry.

### Eligibility criteria

We included all types of full-text publications written in English that reported multiple (> 1) sessions of all types of rTMS in individuals under 26-years-old at enrolment with a psychiatric disorder. We included all types of reports, studies, and multi-session rTMS protocols unless the aim was basic research, protocol development, or to investigate the mechanism of action of rTMS.

### Data extraction and analysis

Three authors (LG, YL and SW) independently screened identified records against the eligibility criteria, extracted the data, and performed the quality assessment. Data extraction was performed with a custom-made form adapted from the Cochrane data collection for intervention reviews ( [32]; see Supplementary Material S1 for details). Any conflicts regarding study eligibility were resolved by discussion. A meta-analysis was not feasible due to significant heterogeneity in study designs, outcome measures, and rTMS protocols.

### Quality assessment

LG, YL and SW independently assessed risk of bias using the Cochrane risk of bias 2.0 tool (RoB 2.0) in randomised controlled trials (RCTs) [33], and the Cochrane tool for risk of bias in non-randomised studies of interventions (ROBINS-I) [34]. Inter-rater agreement was 92%. Conflicts were resolved by discussion.

## Results

**Fig. 1:**
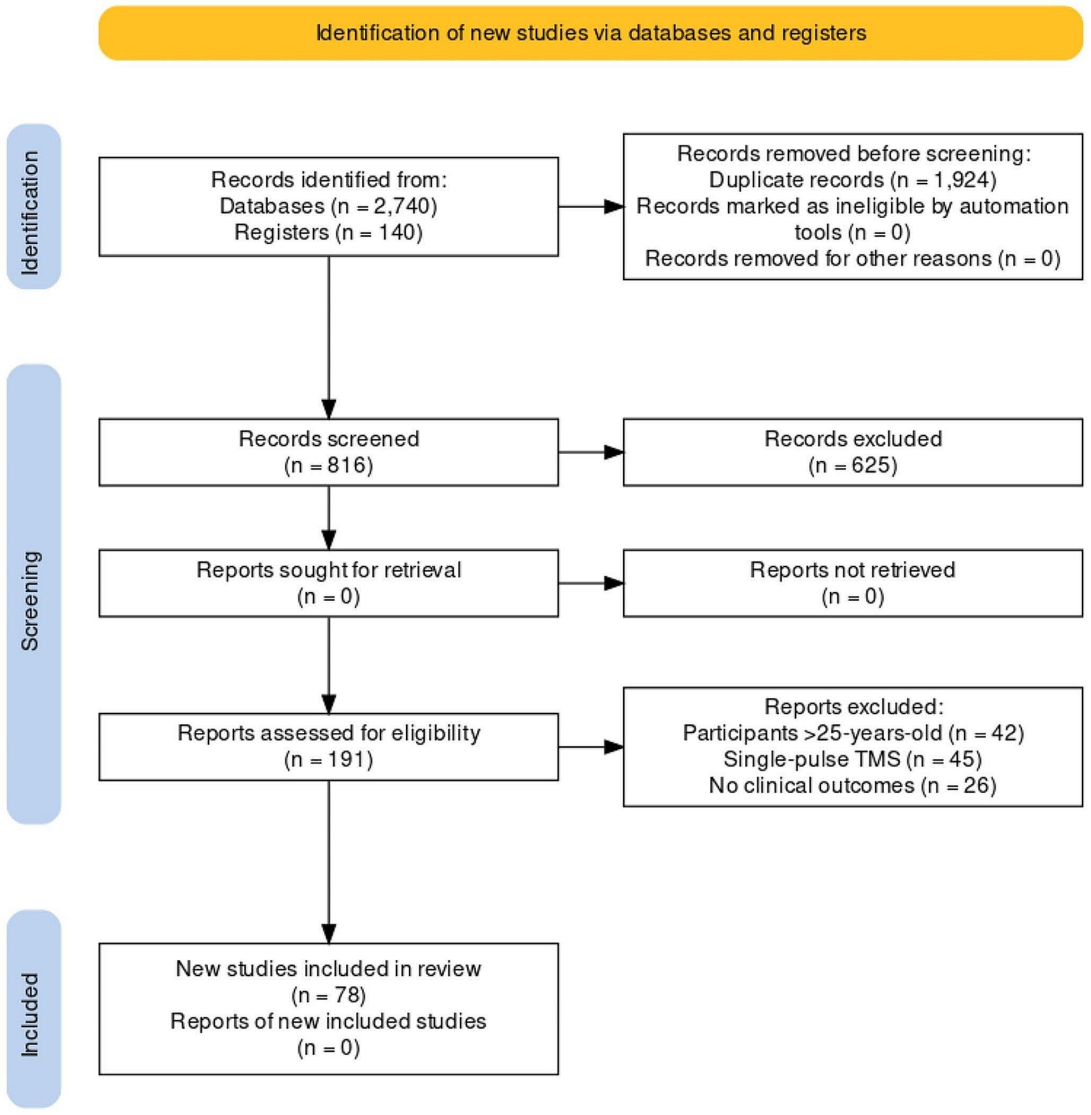
PRISMA flow diagram of selected studies (*n* = number of articles). A total of 78 studies were systematically reviewed

We identified 78 eligible studies (total *N* = 1389; age range 3–25 years, M = 16.43, SD = 4.51; 61.4% male; see Fig. 1), composed of four double-blind, sham-controlled RCTs [35–38], one double-blind, sham-controlled, crossover RCT [39], four single-blind sham-controlled RCTs [40–43], one single-blind, comparator-controlled RCT [44]; one sham-controlled RCT [45], two waitlist-controlled trials [46, 47], one non-randomised, wait-list controlled trial ( [48]; see Table 1), one multi-arm open-label study [49], one two-arm open-label study [50], 27 single-arm open-label studies, and 35 case series/studies (see Supplementary Material S2 and S3). Of these studies involving CYP, 28 studies were in participants with depression, 20 were in ASD, seven in schizophrenia, five in obsessive-compulsive disorder (OCD), four in Tourette’s syndrome, four in attention-deficit/hyperactivity disorder (ADHD), two in anorexia nervosa (AN), borderline personality disorder (BPD), and catatonia, and one in binge eating disorder (BED) and internet gaming disorder (IGD). Across studies, rTMS was typically delivered over ~ 20 sessions (M = 19.7; SD = 8.51; range 5–50) with a stimulation intensity of 80–120% of the resting motor threshold (RMT). The most common protocols employed across studies was 10 Hz, HF-rTMS and/or 1 Hz, LF-rTMS to the left- and/or right dorsolateral prefrontal cortex (DLPFC) (*n* = 44; 56.4%).

**Table 1:** Summary of controlled trials using repetitive transcranial magnetic stimulation in children, adolescents, and young people with psychiatric disorders

Authors (year)	Design	*N*	Diagnosis	Mean age (range)	Stimulation frequency: site of stimulation	Stimulation protocol	Disorder-specific outcome measures	RoB (tool used)	Adverse events (% participants reported)
Chen et al. [40]. ,	Single-blind, randomised, sham-controlled trial	97	MDD	15.0 (12–18)	10 Hz: L-DLPFC	10 sessions; 2400 pulses; 90% RMT	HAM-D; CDRS-R	Some concerns (RoB 2)	Measured, not reported
Croarkin et al. [35]. ,	Double-blind, randomised, sham-controlled trial	103	MDD	(12–21)	10 Hz, HF: L-DLPFC	30 sessions; 3000 pulses; 120% RMT	HAM-D; MADRS; CDRS-R; QIDS-A_17_-SR-I;	Low (RoB 2)	Active vs. Sham rTMS: Eye pain (6% vs. 0%) Nausea (11% vs. 5%) Vomiting (6% vs. 3%) Site pain (4% vs. 0%) Stomach flu (4% vs. 0%) Neck pain (6% vs. 5%) Facial twitching (7% vs. 2%) Limb twitching (4% vs. 0%) Headache (32% vs. 17%) Insomnia (4% vs. 0%) Panic Attacks (4% vs. 0%) Suicidal Ideation (4% vs. 4%)
Gao et al. [41]. ,	Single-blind, randomised, sham-controlled trial	55	MDD	16.8 (13–22)	1 Hz, LF: R-DLPFC	10 sessions; 1000 pulses; 80% RMT	HAM-D; SDS; ANSSIQ	Some concerns (RoB 2)	Not measured or reported
Kaokhieo et al. [36]. ,	Double-blind, randomised, sham-controlled trial	15	ASD	9.0 (7–12)	5 Hz, HF: R-IFG	10 sessions; n/r; n/r	VABS-receptive; VABS-expressive; VABS-written; VABS-communication; VABS-personal; VABS-domestic; VABS-community; VABS-living; VABS-interpersonal; VABS-play; VABS-coping; VABS-socialisation	Some concerns (RoB 2)	Reported ‘no AEs’
Kang et al. [45]. ,	Randomised, sham-controlled trial	32	ASD	7.8	1 Hz, LF: L- & R-DLPFC	18 sessions; 180 pulses; 90% RMT	ABC^1^; ABC^1^-S; ABC^1^-SR; ABC^1^-BO; ABC^1^-L; ABC^1^-S	High (RoB 2)	Not measured or reported
Ni et al. [42]. ,	Single-blind, randomised, sham-controlled trial	75	ASD	12.8 (8–17)	50 Hz, iTBS: pSTS	16 sessions; 600 pulses; 80% AMT	SRS; RBS-R^(a)^	Some concerns (RoB 2)	*Active vs. Sham iTBS Pain at stimulation site (10% vs. 0%) Tinnitus (0% vs. 0%) Headache (3% vs. 0%) Anxiety (0% vs. 3%)
Sokhadze et al. [48]. ,	Non-randomised, waitlist-controlled trial	42	ASD	14.6 (10–21)	1 Hz, LF: L- & R-DLPFC	18 sessions; 180 pulses; 90% RMT	ABC-I; ABC-L; ABC-H; ABC-ST; ABC-SP; RBS-R-total; RBS-R-ST; RBS-R-SI; RBS-R-C; RBS-R-R; RBS-R-RN	Moderate (ROBINS-I)	Not measured or reported
Sokhadze et al. [46]. ,	Waitlist-controlled trial	54	ASD	14.5 (9–21)	1 Hz, LF: L- & R-DLPFC	18 sessions; 180 pulses; 90% RMT	ABC-I; ABC-L; ABC-H; ABC-ST; ABC-SP; RBS-R-total; RBS-R-ST; RBS-R-SI; RBS-R-C; RBS-R-R; RBS-R-RN	Some concerns (RoB 2)	Not measured or reported
Casanova et al. [47]. ,	Waitlist-controlled trial	45	ASD	13.0 (9–19)	1 Hz, LF: L- & R-DLPFC	12 sessions; 150 pulses; 90% RMT	ABC-I; ABC-H; RBS-R	High (RoB 2)	Not measured or reported
Nagy et al. [37]. ,	Double-blind, randomised, sham-controlled trial	60	ADHD	8.6 (6–12)	10 Hz, HF: R-DLPFC	15 sessions; 2000 pulses; 90% RMT	CGI; CPRS-R: L; DSM-IV	Some concerns (RoB 2)	Not measured or reported
Cao et al. [44]. ,	Single-blind, randomised, comparator-controlled trial	64	ADHD	8.5 (6–13)	10 Hz, HF: R-DLPFC	30 sessions; 2000 pulses; 100% RMT	SNAP-IV; CPT	Some concerns (RoB 2)	Reported “apart from mild scalp discomfort and headache, we did not observe any significant side effects”
Weaver et al. [39]	Double-blind, randomised, sham-controlled, crossover trial	9	ADHD	18.1 (14–21)	10 Hz, HF: R-DLPFC	10 sessions; 2000 pulses; 100% RMT	ADHD-IV; CPT	Low (RoB 2)	Mild headache and scalp discomfort (33%)
Wu et al., [38]	Double-blind, randomised, sham-controlled trial	12	TS	14.5	30 Hz, cTBS: SMA	8 sessions; 600 pulses; 90% RMT	YGTSS; RVTRS; PUTS	Low (RoB 2)	Abdominal pain, headaches, and dry eyes (25%)
Pathak et al. [43]	Single-blind, randomised, sham-controlled trial	26	Mania	15.4 (12–17)	20 Hz, HF: R-DLPFC	10 sessions; 800 pulses; 110% RMT	YMRS	Some concerns (RoB 2)	Real vs. Sham rTMS Transient headache (15.4% vs. 0%)

*NOTE*: *AEs assessed actively, i.e., using a questionnaire. All other studies assessed AEs passively – i.e., via spontaneously reported feedback; ^(a)^ = only significant after 8 weeks of iTBS, not at 4 weeks; Green = improvement; Red = no improvement; MDD = Major Depressive Disorder; ASD = Autism Spectrum Disorder; ADHD = Attention Deficit-Hyperactivity Disorder; TS = Tourette’s Syndrome; L = Left; R = Right; iTBS = Intermittent Theta Burst Stimulation; cTBS = Continuous Theta Burst Stimulation; HF = High Frequency rTMS; LF = Low Frequency rTMS; DLPFC = Dorsolateral Prefrontal Cortex; pSTS: Poster Superior Temporal Sulcus; SMA = Supplementary Motor Area; RMT = Resting Motor Threshold; CDRS-R = Children’s Depression Rating Scale-Revised; HAM-D = Hamilton Depression Rating Scale; MADRS = Montgomery-Åsberg Depression Rating Scale; QIDS-A_17_-SR-I = The Quick Inventory of Depressive Symptomatology Adolescent Version; ANSSIQ = Adolescent Non-Suicidal Self-Injury Assessment Questionnaire; SDS = Zung Self-Rating Depression Scale; ABC^1^ = Autism Behaviour Checklist; ABC^1^-SR = Autism Behaviour Checklist – Social Relating; ABC^1^-S = Autism Behaviour Checklist - Sensory; ABC^1^-BO = Autism Behaviour Checklist – Body & Object; ABC^1^-L = Autism Behaviour Checklist – Language; ABC^1^-SA = Autism Behaviour Checklist – Social & Adaptive; SRS = Social Responsiveness Scale; ABC = Aberrant Behaviour Checklist; ABC-I = Aberrant Behaviour Checklist – Irritability; ABC-L = Aberrant Behaviour Checklist – Lethargy; ABC-H = Aberrant Behaviour Checklist – Hyperactivity; ABC-ST = Aberrant Behaviour Checklist – Stereotypy; ABC-SP = Aberrant Behaviour Checklist - Speech; RBS-R = Repetitive Behaviour Scale – Revised; RBS-R-ST = Repetitive Behaviour Scale – Revised – Stereotypy; RBS-R-SI Repetitive Behaviour Scale – Revised – Self-Injury; RBS-R-C = Repetitive Behaviour Scale – Revised – Compulsivity; RBS-R-R = Repetitive Behaviour Scale – Revised – Rituals; RBS-R-RN = Repetitive Behaviour Scale – Revised – Restriction; SNAP-IV = The Swanson, Nolan, and Pelham Rating Scale for ADHD; CPT = Continuous Performance Test for Diagnosis of ADHD; ADHD-IV = ADHD Rating Scale-IV; YGTSS = Yale Global Tic Severity Scale; RVTRS = Rush Video-Based Tic Rating Scale; PUTS = Premonitory Urge for Tics Scale; YMRS = Young Mania Rating Scale; AE = Adverse Event; n/t = not tested; n/r = not reported

### Quality assessment

Of the thirteen RCTs, overall risk-of-bias was rated as “high” in two studies [45, 47]; nine with “some concerns” [35–37, 40–44, 46], and three with “low” [35, 38, 39] (see Supplementary Material S1). The non-randomised, controlled clinical trial was rated with moderate risk of bias [48]. All open-label studies, case series, and case reports were rated as low quality. One RCT was retrospectively registered [37] and four were prospectively registered [35, 38, 40, 42] (and comparison of the registered protocol with final publication showed that one study omitted two registered primary outcomes ( [42]; autism spectrum quotient and social communication questionnaire). One RCT [36] reported an incorrect clinical trial registry identifier and we were unable to find the study record using other trial information (e.g., investigator name) in the search function.

### What are the clinical effects of rTMS in children and young people with psychiatric disorders?

#### Neurodevelopmental disorders

##### Autism spectrum disorder (ASD)

There have been six controlled trials conducted in ASD. In an integrated 4-week RCT and 4-week open-label extension study [42], iTBS was delivered to the bilateral posterior superior temporal sulcus in 78 CYP with ASD. During the first four weeks, the study group received two sessions of iTBS per week (8-sessions total) and the control group received sham TBS. After unblinding, both groups received eight sessions of real iTBS over the next four weeks. Results showed there were no significant group-by-time interactions for the study vs. control group on clinical symptoms in the first four or eight weeks. However, within-group analysis showed significant improvements in parent-rated social skills and repetitive behaviours at week eight compared to baseline for the 8-week iTBS group [42].

One double-blind RCT [36] applied 10 sessions of HF-rTMS to the right inferior frontal gyrus combined with action observation and execution (AOE) training or sham rTMS with AOE to 10 CYP with ASD. Within-group analyses showed a significant increase in clinician-rated receptive and expressive communication, as well as domestic and community daily living, from baseline to 1-week follow-up in the group that received HF-rTMS + AOE. In addition, clinician-rated communication significantly increased in the sham rTMS + AOE and the HF-rTMS + AOE groups from baseline to 1-week follow-up [36].

A randomised, sham-controlled trial [45] in 32 children with ASD and intellectual disability, reported significant reductions in parent-rated social relating behaviours, and overall non-adaptive behaviours, immediately after 18-sessions of LF-rTMS to the left- and right-DLPFC, compared to baseline. Although no significant changes were reported in the sham-group, no between-group analysis was conducted. One randomised waitlist-controlled study [47] in 45 adolescents with ASD reported significant between-group differences in parent-rated repetitive and restricted behaviour patterns and irritability immediately after 12-sessions of LF-rTMS, but no difference in social awareness and hyperactivity. These authors [47] did not report outcomes for any other subscales.

A non-randomised waitlist-controlled study [48] in 42 children with ASD reported a significant group-by-time interaction in parent-rated total repetitive and restrictive behaviours, lethargy, and hyperactivity, but no change in irritability, stereotypy, inappropriate speech, self-injurious behaviour or restricted interests, due to a significant decrease from baseline after 18-sessions of LF-rTMS to the right-DLPFC combined with neurofeedback, with no change in the waitlist group. Another waitlist-controlled study (no randomisation details provided; [46]) in 54 adolescents with ASD, reported significant improvements in parent-rated stereotypic behaviour, ritualistic behaviour, irritability, lethargy, compulsivity, and hyperactivity, but no change in inappropriate speech, self-injurious behaviour, and restricted interests, after 18-sessions of LF-rTMS to the right-DLPFC: no changes were seen in the waiting-list group, compared to baseline.

This research group also conducted one multi-arm open-label study [49] and six single-arm open-label studies [51–56]. These studies evaluated several outcomes (e.g., Visual oddball task, Aberrant Behaviour Checklist, Repetitive Behaviour Scale-Revised), with some reporting improvement and other outcomes showing no change (see Table 2). The authors provided no evidence to show these studies were statistically powered to detect change and they did not correct for multiple comparisons.

Seven additional open-label studies or case studies were conducted in ASD. Two open-label studies applied HF-rTMS to the inferior parietal lobule (IPL) [57, 58]. One study reported significant improvements in parent-rated speech/language and sociability from baseline to 6-weeks after 30-sessions of HF-rTMS, but not from baseline to post-stimulation, in 11 children with low-functioning ASD [58]. The other study, in four children with ASD, reported no significant changes in clinician-rated ASD symptoms, or parent-rated sociability and communication, immediately after 9-sessions of rTMS compared to baseline [57].

One open-label study [59] applied 19 sessions of individual alpha frequency (IAF)-guided rTMS to 28 children with ASD. IAF-guided rTMS was determined by identifying the dominant peak frequency with the highest power in the 8–13 Hz range and multiplying it by the higher harmonic frequency (5th to 10th ) of the electroencephalogram nearest to the dominant peak frequency. The stimulation site was determined by identifying the brain region with the highest aberrant cortical processes compared to a normative database with equal parameters and measured using the 10–20 system. Within-group analyses showed significant improvement in clinician-rated emotional response, object use, fear or nervousness, intellectual response, and general impressions of autism from baseline to post-treatment [59].

Another open-label study [60], applied 15-sessions of iTBS to the right-DLPFC in 10 adolescents with ASD and reported significant reductions in parent-rated restricted and repetitive behaviour, and obsessive-compulsive symptoms, from baseline to immediately post-stimulation.

A case study [61] in a 15-year-old male with ASD and comorbid depression described improvements in mood and core features of ASD following 20 sessions of LF-rTMS to the right-DLPFC and 10-sessions of LF-rTMS to the left-DLPFC. Two case studies applied deep-TMS ?? to the medial-PFC: in one study [62] a 20-year-old woman with high-functioning ASD, self-reported improvements in social functioning following 9-sessions of deep-TMS, and the other study [63] reported improvements in clinician-rated obsessive-compulsive symptoms, but not self-reported autistic traits, in a 25-year-old female with ASD and comorbid OCD immediately after 27 sessions of deep-TMS.

##### Attention deficit hyperactivity disorder (ADHD)

Three controlled trials applied HF-rTMS to the right-DLPFC in CYP with ADHD. In a double-blind, sham-controlled RCT [37] in 60 CYP with ADHD, atomoxetine combined with 15 sessions of active HF-rTMS significantly improved parent-rated total ADHD symptoms and teacher-rated inattention at post-treatment and 1-month follow-up compared to sham, as well as parent-rated hyperactive-impulsivity at post-treatment, but not at 1-month follow-up [37]. In a multi-arm, active-comparator RCT [44], 60 children with newly diagnosed ADHD were randomly assigned to receive (a) 30 sessions of HF-rTMS over 6 weeks, (b) atomoxetine once-daily over 6 weeks, (c) HF-rTMS and atomoxetine over 6 weeks. All three groups showed significant improvements in parent-rated severity of core ADHD symptoms at post-stimulation compared to baseline, but the group that received HF-rTMS and atomoxetine showed significantly greater change scores for attention deficit, hyperactivity and impulsivity, but not oppositional defiance, compared to the groups that received HF-rTMS or atomoxetine alone [44]. A sham-controlled, crossover RCT [39] in nine adolescents with ADHD reported no significant differences in core ADHD symptoms or clinical global impression between 10-sessions of real or sham HF-rTMS to the right-DLPFC.

An open-label study [63] applied 5-sessions of LF-rTMS over the left-DLPFC in 13 boys with ADHD and reported a significant improvement in parent- and teacher-rated behaviour at home and at school from baseline to 1-week post-stimulation.

##### Tourette’s syndrome

Four studies applied LF-rTMS or cTBS to the supplementary motor area (SMA) in CYP with Tourette’s syndrome. A randomised, sham-controlled trial [38] delivered 8-sessions of cTBS in 4 trains per day over 2 consecutive days in 12 adolescents with chronic tics. No significant differences in clinician-rated severity of tic symptoms and obsessive-compulsive symptom severity, self-reported severity of tic-related premonitory urges and health-related quality of life, or parent-rated ADHD symptom severity were found between the real and sham cTBS group at 7-days follow-up [38]. An open-label study [64] in 10 children with Tourette’s syndrome reported a significant reduction in clinician-rated tic severity and impact of tics on quality of life after 10-sessions of LF-rTMS, and at 12-week follow-up, compared to baseline. Another open-label study [65] applied 15-sessions of bilateral LF-rTMS to nine CYP with Tourette’s syndrome and reported that tic severity significantly decreased from baseline to post-treatment. Lastly, a case study [66] applied 10-sessions of LF-rTMS in two CYP with Tourette’s syndrome and reported improvement in clinician-rated tic severity from baseline to post-stimulation which was maintained for 1-month in one case and 4-months in the other case. After 1-month, the first case had recurring tic symptoms and 10 additional sessions of LF-rTMS were delivered with similar improvement in clinician-rated tic severity reported [66].

#### Schizophrenia-Spectrum disorders

##### Schizophrenia

Four studies applied LF-rTMS to the left temporoparietal cortex (TPP) in CYP with schizophrenia. One open-label study [67] in 10 adolescents with schizophrenia reported a significant improvement in auditory hallucinations and psychosocial functioning immediately after, and 1-month after, 10-sessions of twice-daily LF-rTMS compared to baseline. In a case study in an 11-year-old boy with schizophrenia [68], 10-sessions of LF-rTMS to the right-temporoparietal junction (TPJ) was not associated with improvements in auditory hallucinations compared to baseline, but after 10-sessions of LF-rTMS to the left-TPJ, auditory hallucinations reduced, and this improvement was maintained at 6-week follow-up [68].

In another case study [69], an 18-year-old female with schizophrenia received 3 separate courses of 10- and 15-sessions LF-rTMS to the left-TPP due to improved self-reported reduced auditory hallucinations following rTMS and subsequent recurrence of symptoms at 5-months after the first course of rTMS and 3-months after the second course of rTMS. After the third course of rTMS, the patient self-reported maintained improvement in auditory hallucinations at 4-months [69]. Similarly, another case study [70] applied two separate courses of 10-sessions of LF-rTMS over the left-TPP in an 18-year-old woman with schizophrenia who self-reported minimal change in auditory hallucinations during the first course of rTMS and progressive improvement during the second course of LF-rTMS with complete cessation of auditory hallucinations at post–stimulation and 7-months follow-up [70].

In a case study [71], a 22-year-old male with schizophrenia self-reported a reduction in the frequency, attentional salience, and distress level of auditory hallucinations, but no change in loudness and length, over the course of 20-sessions of LF-rTMS to the left superior temporal gyrus (STG), but the frequency of hallucinations returned to baseline level at 1-week follow-up [71]. Lastly, a case study [72] reported reduced clinician-rated negative symptoms after 20-sessions of HF-rTMS to the left-DLPFC in an 18-year-old female with schizophrenia. However, after 18-sessions, the patient also became overtly concerned about hygiene and started on fluoxetine following a diagnosis of OCD which improved symptoms and this improvement was maintained, alongside improvements in negative symptoms, at 6-months after HF-rTMS [72].

##### Catatonia

Two case studies applied HF-rTMS to the left-DLPFC in adolescents with catatonia: the first study [73] reported improvement in catatonic symptoms, (including verbal output, mobility, mutism, and poor eye contact), after 19-sessions of HF-rTMS and at 1-year follow-up in a 16-year-old female [73]. The second study [74], reported a near complete resolution of catatonic symptoms after 10-sessions of HF-rTMS, and at 3-days follow-up, in an 18-year-old female.

#### Mood disorders

##### Major depressive disorder (MDD)

The largest double-blind, sham-controlled RCT [35] to date, applied 30-sessions of real or sham HF-rTMS to the left-DLPFC in 103 adolescents with treatment-resistant depression (TRD). No significant differences were reported for change scores in clinician-rated depression severity from baseline to post-stimulation, or in response and remission rates at post-stimulation, between the real and sham rTMS groups. In another large RCT [40], 100 adolescents were randomly assigned to receive 10 sessions of HF-rTMS to the left-DLPFC and 50 mg of sertraline (study group) or just 50 mg of daily sertraline (control group) over two weeks. Both groups continued with sertraline in the two weeks following rTMS, but participants with < 50% HAM-D reduction in the first two weeks, were administered 100 mg of sertraline daily. Responder rates were significantly higher for the study group compared to the control group after 2 and 4 weeks. In addition, the study group had significantly lower HAM-D and CDRS-R scores at the end of week two and four compared to the control group [40].

In a single-blind, sham-controlled RCT [41], 55 adolescents with depression were grouped according to medication use before randomisation to receive 10-sessions of real LF-rTMS to the right-DLPFC, or sham. Compared to sham, the medicated and non-medicated real rTMS groups showed a significant reduction in clinician-rated and self-reported depression severity at post-stimulation. The non-medicated real rTMS group also reported significantly fewer self-injury impulses and thoughts compared to the non-medicated sham group at post-stimulation [41].

A single-blind randomised study [preprint; 49] delivered 20 sessions of either LF- or HF-rTMS to the right- or left-DLPFC, respectively, to 14 CYP with MDD. As findings from the random effect regressions demonstrated that HF-rTMS and LF-rTMS groups did not differ on CDRS-R scores over time, the groups were collapsed and the whole sample was reported together. There was a significant improvement in CDRS-R scores from baseline to after 10 sessions, after 20 sessions, and at 1-month follow-up. Mean CDRS-R scores also significantly declined from baseline to 6-month follow-up. Immediately after 20 sessions, only one participant achieved partial response (25–50% reduction in CDRS-R score), with none of the CYP achieving *≥* 50% reduction in CDRS-R, but at 1-month follow-up, two CYP achieved *≥* 50% reduction in CDRS-R scores, and four CYP achieved partial response [50].

Two studies assessed the age-dependent effects of rTMS. One study [75] applied 18-sessions of LF-rTMS to the right-DLPFC (*n* = 11) or bilateral HF-rTMS to the left-DLPFC and LF-rTMS to the right-DLPFC (*n* = 4) in 15 young adults with TRD. Clinician-rated and self-reported depression severity reduced significantly from baseline to post-stimulation and response rates did not significantly differ from data in adults aged 25–82 (*n* = 229). The other study [76] compared the effects of > 15 sessions (*M* = 15.6) of add-on HF-rTMS to the left-DLPFC in 42 adolescents, 27 adults, and 48 older adults with depression and reported that significantly more adolescents met remission criteria than adults and older adults at week-2 of rTMS, and older adults at week-4 [76]. However, the adolescent group had significantly lower baseline clinician-rated depression severity than the adult and older adult groups. Post-hoc, within-group analysis also revealed a significant reduction in somatic and psychic anxiety in all age groups at week-2 and week-4 of rTMS compared to baseline, however this was not compared between age groups [77]. This research group also pooled data to compare the effects of 10 sessions of HF-rTMS to the left-DLPFC or LF-rTMS to the right-DLPFC on suicidal ideation between adolescents and adults with depression [78]. Significantly more adolescents than adults achieved suicidal ideation remission at post-stimulation and adolescents who received HF-left-DLPFC rTMS were more likely to achieve remission than adolescents who received LF-right-DLPFC rTMS [78].

Six open-label studies applied HF-rTMS to the left-DLPFC in adolescents with depression. Two studies applied 30-sessions of rTMS in eight [79] and ten [80] adolescents with depression, with both studies reporting a significant reduction in clinician-rated depression severity score, and clinician-rated illness severity in Wall et al. [80]. , , from baseline to session-10, session-20, post-stimulation, and 6-months post-stimulation. Self-reported depression severity also significantly reduced from baseline to session-20, post-stimulation and 6-months post-stimulation [80]. Two post-hoc studies pooled data from Wall et al. [79–81]. , with one reporting improvements in suicidal ideation over the course of rTMS which was mediated by improvement in depressive symptom severity [82] and the other reporting a significant improvement in hypersomnia, but not insomnia, from baseline to session-10 and 6-month follow-up [83].

Two studies have applied 15-sessions of rTMS: one study [84] found a significant reduction in clinician-rated depression and anxiety symptoms, and self-reported depression severity, from baseline to post-stimulation and a 56% response rate in 32 adolescents with TRD. The other study [85] reported a reduction in clinician-rated depression and anxiety symptoms, and self-reported depression symptoms, from baseline to post-stimulation and a 67% response rate in six adolescents with depression.

The last open-label study [86], applied 14-sessions of rTMS in nine adolescents with depression. Compared to baseline, clinician-rated and self-reported depression symptoms reduced significantly at day-7, day-10, and 1-month follow-up, whereas self-reported anxiety symptoms reduced significantly only at post-stimulation and 1-month follow-up and clinician-rated illness severity reduced significantly from baseline to post-stimulation only. No significant changes were found in suicidal ideation [86]. A follow-up study [87] with eight of nine participants from Bloch et al. [86]. , revealed no significant difference in self-reported and clinician-rated depression from 1-month follow-up to 3-year follow-up, but five participants were categorised in the minimal range of depression severity compared to only one participant in this range at 1-month follow-up.

One open-label study [88] applied 10-sessions of bilateral intermittent theta burst stimulation (iTBS) to the left-DLPFC and continuous TBS (cTBS) to the right-DLPFC in 20 adolescents and young people with depression. Clinician-rated and self-reported depression symptoms, and quality of life, significantly improved from baseline to session-5 and post-stimulation, with a 20% response rate and 10% achieving remission [88]. In the absence of a control-group, placebo-effects cannot be ruled out in any of these open-label studies [79–88].

Several case studies reported improvements following rTMS. A case series [89] applied 7-sessions of HF-rTMS to the left-DLPFC in 3 adolescents aged 15-, 16-, and 17-years-old and clinician-rated depression symptoms and self-reported suicidal ideation reduced from baseline to post-stimulation. However, symptoms of hypomania occurred in two participants from day-4 of stimulation, one of whom received a diagnosis of hypomania that was resolved by a change of medication [89]. Another case series [90] in two 16-year-old females, reported improvement in clinician-rated and self-reported depression symptoms, and overall illness severity, after > 25-sessions of HF-rTMS to the left-DLPFC compared to baseline. Clinician-rated illness severity and depression symptoms were assessed at 1-month follow-up and improvements were maintained in both participants [90].

In a case study [91], a 17-year-old male with depression showed fewer anxiety symptoms at 1-, 2-, and 4-weeks after 20-sessions of HF-rTMS to the left-DLPFC but showed no change in depression or suicidality scores. In another case study [92], a 23-year-old female with depression and ADHD, self-reported improvement in depression symptoms at session-3, post-stimulation, and 14-months after receiving 13-sessions of concurrent LF-rTMS applied to the anterior cingulate cortex with intravenous ketamine infusions. Lastly, a 24-year-old male with TRD and comorbid PTSD who received 22-sessions of HF-rTMS to the left-DLPFC showed a reduction in self-reported suicidal ideation, PTSD and depression symptoms at 1- and 3-week follow-up [93].

##### Bipolar mania

A single-blind, randomised, sham-controlled trial [43] in 26 adolescents with bipolar mania applied 10-sessions of HF-rTMS to the right-DLPFC and reported no significant differences in clinician-rated severity of manic symptoms and overall severity of illness between the real and sham rTMS groups.

#### Obsessive compulsive disorder (OCD)

Two case studies applied LF-rTMS to the supplementary motor area (SMA). One study [94] reported improvement in clinician-rated severity of obsessive-compulsive symptoms and ritualistic behaviours after 35-sessions of LF-rTMS (5-sessions per week) and after two phases of maintenance LF-rTMS (21-sessions, 2-sessions per week; 23-sessions, 1-session per week) when symptoms re-emerged in a 23-year-old woman with OCD at 2- and 8-weeks after the first and second course of LF-rTMS finished. Another case study [95] reported improvement in clinician-rated severity of obsessive-compulsive symptoms following 20-sessions of LF-rTMS, and improvement in daily functioning at 3-months post-stimulation, in a 19-year-old man with OCD, with no maintenance rTMS required in the follow-up period. Another case study [96] applied 30-sessions of dual-site LF-rTMS to the SMA and orbitofrontal cortex and reported improvements in clinician-rated severity of obsessive-compulsive symptoms and overall severity of illness at post-stimulation compared to baseline in an 18-year-old female with OCD and comorbid depression.

Another case study [97] applied 27-sessions of add-on neuronavigated iTBS to the pre-SMA in a 21-year-old male with OCD secondary to a cerebellar lesion and reported a reduction in clinician-rated severity of obsessive-compulsive symptoms at session-10, post-stimulation and at 3-months follow-up, after which, symptoms gradually reverted to baseline (pre- intervention) severity.

#### Eating disorders

Three case studies applied HF-rTMS to the left-DLPFC in anorexia nervosa (AN) [98, 99] or binge eating disorder (BED) [99]. One case study [98] in a 23-year-old female with AN reported improvement in body mass index (BMI), laxative and diuretic abuse, and attitude towards body, weight, shape, and food after 21-sessions of HF-rTMS, and at 8-week follow-up, these improvements were sustained, together with a further increase in BMI. Another case study [99], applied 20-sessions of HF-rTMS in a 23-year-old female and reported improvements in self-rated eating disorder symptoms, but no change in weight, at post-stimulation, and, at 1-month follow-up, self-rated eating disorder symptoms had further improved to below the clinical cut-off score. Finally, a case study [100] applied 20-sessions of HF-rTMS in a 19-year-old female with BED and comorbid depression who reported no binge eating episodes during the last 2-weeks of HF-rTMS, as well as improvements in binge eating symptoms and clinician-rated overall severity of illness at post-stimulation.

#### Personality disorder

A case series [101] applied 15-sessions of HF-rTMS in a 20- and 23-year-old female and a 23-year-old male with borderline personality disorder (BPD) and reported improvement in overall severity of illness in all 3 participants at post-stimulation, compared to baseline. A case study [102] in a 22-year-old female reported improvements in self-reported impulsivity and clinician-rated severity of BPD, immediately after 10-sessions of HF-rTMS compared to baseline.

#### Social anxiety disorder (SAD)

A case study [103] in a 23-year-old male with SAD reported reductions in self-reported anxiety and social anxiety symptoms immediately after 12-sessions of LF-rTMS to the right-ventromedial prefrontal cortex, and at 2-weeks follow-up, compared to baseline.

#### Internet gaming disorder (IGD)

A case study [104] in a 21-year-old male with IGD self-reported improvements in depressive symptoms, current craving for gaming, internet gaming disorder symptoms, and internet addiction symptoms, but not self-rated overall severity of symptoms, anxiety symptoms, or self-rated sleep quality, 3-weeks after 26-sessions of HF-rTMS to the left-DLPFC. Improvements in self-reported depressive symptoms, current craving for gaming, internet gaming disorder symptoms, and internet disorder symptoms had increased further at 1-year follow-up. Self-rated sleep quality and anxiety symptoms both worsened at 3-week follow-up but had improved to near baseline levels by 1-year follow-up [104].

### In children and young people with disorders other than mood disorders, what are the effects of rTMS on mood?

None of the studies assessed the effects of rTMS on mood in CYP with ASD, schizophrenia, ADHD, Tourette’s syndrome, and catatonia.

#### OCD

One case study [96] in an 18-year-old female with OCD and comorbid depression, reported a reduction in clinician-rated severity of depressive symptoms following 30-sessions of LF-rTMS to the SMA and OFC compared to baseline.

#### Eating disorders

One case study [99] in a 23-year-old female with BED reported a reduction in self-rated depression, anxiety, and stress symptoms immediately after 20-sessions of HF-rTMS to the left-DLPFC, but at 1-month follow-up, all three subscale scores had increased, although anxiety and stress subscale scores remained lower than baseline. Another case study [100] in a 19-year-old female with BED, and with comorbid depression, reported improvements in parent- and self-reported mood and depressive symptoms immediately after 20 sessions of HF-rTMS to the left-DLPFC.

#### Personality disorder

A case series [101] in three young people with BPD, reported improvements in clinician-rated severity of depressive symptoms immediately after receiving 15-sessions of HF-rTMS to the right-DLPFC. A case study [102] reported a decrease in self-reported depressive symptoms immediately after 10-sessions of HF-rTMS to the left-DLPFC and at 1-month follow-up, but not at 3-months follow-up, and improvements in self-reported negative affect at 1-month follow-up, but not immediately post-stimulation or at 3-month follow-up.

#### Social anxiety disorder

One case study [103] in a 23-year-old male, reported improvements in self-reported depressive symptoms immediately after 12-sessions of LF-rTMS to the right-vmPFC.

### What are the effects of rTMS on neurocognition in this population?

No studies assessed the effects of rTMS on neurocognition in CYP with social anxiety disorder, bipolar mania, personality disorder, substance abuse disorder, catatonia, eating disorders, OCD or Tourette’s syndrome.

#### Neurodevelopmental disorders

##### ASD

One research group has published seven studies that have employed a Kanizsa figures three-stimuli oddball task, with all bar one [105], reporting a significant reduction in total error rate and all reporting post-error normative reaction time slowing in adolescents with ASD following 12- or 18-sessions of LF-rTMS to the left- and right-DLPFC, compared to baseline [54] or waiting-list group [46–49, 47, 105]. In addition, two studies reported significant reductions in omission error rates, but no difference in commission error rates [47, 105], whereas other studies reported significant reductions in commission error rates, but no difference in omission error rates [46–49, 54].

An open-label study [60] in 10 boys with ASD, reported significantly fewer perservative errors and improved performance on the Wisconsin Card Sorting Test (WCST), as well as significant reductions in total time taken to complete the Stroop test, immediately after 15-sessions of neuronavigated iTBS to the right-DLPFC, compared to baseline. Lastly, a case study [63] in a 25-year-old female with high-functioning ASD and comorbid OCD, reported improvements in executive function, attention, speed processing, visual spatial, and motor skills, but not memory or verbal function, immediately after 27-sessions of deep-TMS to the medial-PFC.

##### ADHD

A multi-arm, active-comparator RCT [44] in 60 children with newly diagnosed ADHD, reported a significant improvement in auditory and visual attention and working memory, and decision-making on the Iowa Gambling Task, in the HF-rTMS, atomoxetine, and combined HF-rTMS and atomoxetine group. The combined HF-rTMS and atomoxetine group showed significantly greater improvements in decision-making, and visual and auditory attention and working memory compared to rTMS only group and atomoxetine only.

#### Depression

An open-label study [81] applied 30-sessions of HF-rTMS to the left-DLPFC in 14 adolescents with major depressive disorder and reported an improvement in immediate memory and delayed recall, but no change in interference, immediate recall, level of learning, or verbal and non-verbal executive function, at post-stimulation, compared to baseline. Another open-label study [86] in 9 adolescents with depression reported improvements in reaction time immediately after 14-sessions of HF-rTMS to the left-DLPFC, and at 1-month follow-up, as well as improvements in planning at 1-month follow-up, only. Eight out of nine participants participated in a 3-year follow-up study [87] which found no evidence of deterioration in cognitive functioning from 1-month to 3-year follow-up.

A case study [91] in a 17-year-old male with major depressive disorder, reported no significant changes in cognitive impairment, memory, and learning, working memory and executive function, visual memory, attention and reaction time, semantic memory, decision-making and response control immediately after 20-sessions of HF-rTMS to the left-DLPFC, or at 1-, 2-, and 4-week follow-up. Another case study [90] in two 16-year-old females with depression, reported no change in attention and concentration, executive function, or memory and psychomotor speed immediately after ~ 30-sessions of HF-rTMS to the left-DLPFC, or at 1-month follow-up.

#### Schizophrenia

A case study [67] in an 11-year-old boy with schizophrenia reported a significant improvement in source-monitoring capacity, due to a significant reduction in false internal and external attributions immediately after 10-sessions of LF-rTMS to the left- and right-TPJ, compared to baseline.

### What is the safety profile of rTMS in children and young people with psychiatric disorders?

Overall, rTMS was well-tolerated and feasible in a variety of age groups and psychiatric disorders, which extends existing evidence of a benign side-effect and good tolerability profile of rTMS in CYP [106]. However, adverse events (AEs) were not measured or reported in 40 studies and whilst five studies reported no AEs, it was not clear whether sensory side effects were measured [36, 57, 60, 65, 67]. Only four studies reported monitoring AEs actively (i.e., using a structured questionnaire that lists specific AEs), whereas the remaining studies monitored AEs passively (i.e., relying on spontaneous feedback from participants or caregivers; see Table 1, and S2 & S3). Selective reporting bias is very likely as the frequency of AEs increases when monitored actively [107].

Six case studies reported TMS-related seizures in six females aged between 15 to 19-years old with depression [108–111], schizophrenia [112], or OCD with comorbid depression [113], and five studies reported TMS-related, new-onset psychiatric symptoms and/or diagnoses, including suicidality [83], affective switching to hypomania [89, 114], Charles Bonnet Syndrome [115], and OCD [72]. None of these cases were reported to be at increased risk of seizure at the time of stimulation, except for one that was complicated by alcohol-use [108].

### Unpublished registered trials

Of the 17 registered trials (see Supplementary Material S4), six had not started recruiting, one is enrolling by invitation, four are completed, and the remaining six are ongoing. One is a quadruple-blind RCT, four are triple-blind RCTs, six are double-blind RCTs, and six are open-label single-arm studies. These 17 studies are (a) recruiting either ASD (*n* = 5), ADHD (*n* = 1), MDD (*n* = 6), AN (*n* = 2), Tourette Syndrome (*n* = 2), and Tic Disorders (*n* = 1); (b) stimulating the DLPFC (*n* = 6), SMA (*n* = 4), motor cortex (*n* = 3), DMPFC (*n* = 1), OCC (*n* = 1), or SFG (*n* = 1); (c) applying rTMS alone (*n* = 14), or combining stimulation with cognitive training (*n* = 1) or Comprehensive Behavioural Intervention for Tics (*n* = 2); and (d) recruiting ~ 60 participants (range: 15–200) per trial, in CYP aged (on average) between 10 and 18 years old.

## Discussion

This is the first systematic review that synthesises published and unpublished studies investigating the effects of multi-session rTMS in CYP with psychiatric disorders. Thus far, studies are limited to case series/studies (*n* = 65; 83%) and mainly small, randomised, or non-randomised, trials (*n* = 14). Overall, these studies demonstrate that rTMS is well-tolerated across psychiatric disorders in CYP, and that it is feasible to conduct larger-scale RCTs in ASD and depression. Initial evidence is encouraging in terms of clinical, cognitive, or mood outcomes following rTMS, but it is not yet possible to make any strong conclusions on the therapeutic efficacy of rTMS for CYP with psychiatric disorders.

Out of 78 included studies, 62 studies measured clinical effects immediately after the final rTMS session, with all, except eight [35, 39, 43, 50, 57, 58, 63, 91] reporting an improvement in at least one outcome measure of core disorder-specific symptoms. Three studies assessed clinical outcomes at 1-week [38, 116] or 3-weeks [104] after rTMS, two of which [104, 116] reported improvements in disorder-specific symptoms. Of 25 studies that measured clinical effects at a longer-term follow-up, improvements in core symptoms persisted at 3-days [74], 1-week [36, 93], 2-weeks [103], 3-weeks [93], 1-month [37, 66, 67, 73, 86, 90, 99], 6-weeks [68], 2-months [98], 3-months [64, 95, 97], 6-months [72, 79, 80, 83], 7-months [70], 1-year [104], and 14-months follow-up [92]. One study reported that improvements at post-stimulation were not maintained 1-week later [71] and another follow-up study reported that improvements at 1-month [86] were not maintained at 3-years follow-up [87]. Two studies reported improvements in disorder-specific symptoms only at one-month [50] or 6-weeks follow-up [58], but not immediately post-stimulation, indicating a delayed therapeutic effect of rTMS, which is consistent with data in adults with psychiatric disorders [117, 118]. Therefore, future studies should assess core symptoms and related impairments of psychiatric disorders in CYP at post-stimulation and at least one longer-term follow-up point, to capture any delayed improvements.

Overall, these findings suggest that the therapeutic effects of rTMS on disorder-specific symptoms appear to persist post-stimulation in CYP with psychiatric disorders and may offer beneficial long-term effectiveness. Currently, the limited available evidence suggests that rTMS may be most beneficial for CYP with ASD, ADHD, MDD, and schizophrenia. In terms of a hierarchy for the strength of evidence, the evidence is strongest for neurodevelopmental disorders, encompassing ASD, ADHD, and TS – as there is most research, and crucially most randomised, sham controlled trials (three for ASD, two for ADHD, one for TS). These studies demonstrated improvements in communication and social responsiveness in all three trials for ASD [36, 42, 45], global ADHD symptoms and inattention in one [37] of the two trials for ADHD [37, 44], and no significant differences were found in the one trial for TS [38]. Evidence for rTMS in MDD is the second strongest, with three randomised and sham controlled trials [35, 40, 41], and two [40, 41] out of the three finding significant improvements in mood. There was a randomised and sham controlled trial for mania in CYP, which reported no significant improvements [43]. For the remaining disorders for which rTMS has been explored, schizophrenia, OCD, EDs, catatonia, IGD and BPD, there are no available randomised and sham controlled trials conducted in CYP, therefore the existing research does not yet permit distinction between them in terms of the strength of evidence.

Indeed, 49 of 54 studies reporting improvements in disorder-specific symptoms at post-stimulation were open-label studies or case series/studies: therefore, we cannot rule out the possibility that improvements were due to placebo effects. This is important because four out of 10 sham-controlled RCTs reported no significant differences in disorder-specific symptoms between real and sham rTMS, due to improvement in both real and sham rTMS groups [35, 38, 39, 43], with one reporting a 36.4% placebo response rate [35]. This is consistent with evidence in adults, for example, a meta-analysis of sham response magnitudes in 61 RCTs of rTMS for depression reported a large placebo response effect size (g *=* 0.8) and a meta-regression revealed that sham rTMS responses may be increasing over time [119]. Several non-specific factors, regarding the context and delivery of rTMS, are thought to contribute to sham response, including the sophisticated appearance of TMS equipment, the hands-on nature of the procedure, and the lengthy interactions with the individual administering TMS [120]. Although disentangling real and sham effects is particularly difficult in TMS research, it is a necessary consideration for future studies. As evidence shows that placebo response decreases with increasing treatment resistance [121], one way to address this is to recruit a more severe, treatment-resistant, homogenous patient population in RCTs using rTMS in CYP with psychiatric disorders, as this is more likely to separate active and sham response. Alternatively, a placebo run-in period would enable the exclusion of patients who respond well to the placebo intervention.

Only 21 studies measured neurocognitive and mood outcomes. 15 studies measured neurocognitive outcomes, of which 12 reported improvements immediately after rTMS [44, 46–49, 47, 54, 60, 63, 67, 81, 86, 105]. One study found that improvements were maintained at 1-month follow-up (compared to baseline), as well as improvements in neurocognitive outcomes that were non-significant at post-stimulation that became significant at 1-month follow-up compared to baseline [86]. All six studies assessing mood outcomes found improvements post-stimulation [96, 99–103], with one study testing and finding the effect at 1-month follow-up, but not at 3-month follow-up [102], and another reported that effects were not maintained at 1-month follow-up [99].

14 studies reporting improvements in cognition or mood outcomes, stimulated the DLPFC: this is consistent with evidence that rTMS to the DLPFC modulates neuronal networks involved in emotion regulation and executive function [122, 123]. 28 other studies also stimulated the DLPFC but did not measure cognitive or mood outcomes. Low mood and cognitive deficits, particularly in executive control, are common features across psychiatric disorders in CYP [124, 125] and are thought to contribute to illness complexity, burden, and treatment resistance [126]. It is therefore important that future research measures the effects of rTMS on a range of disorder-relevant cognitive impairments and mood outcomes.

Because of the limited and heterogeneous evidence base, small sample sizes and a lack of consistency in the parameters used, it was not possible to identify optimal stimulation parameters for CYP with psychiatric disorders. The effects of rTMS depend on several stimulation parameters, e.g., frequency, stimulation intensity, number of sessions, and site of stimulation, but no dosage guidance exists for rTMS use in CYP. Most of the studies included in this review applied 10–20 sessions of LF- and/or HF-rTMS to the left- and/or right-DLPFC at ~ 100% RMT, which highlights the use of overly general stimulation protocols across psychiatric disorders in CYP that do not account for disorder-specific or interindividual variations in brain characteristics [127]. In addition, generalised stimulation protocols do not account for clinical heterogeneity, e.g., psychiatric comorbidities. The DLPFC is one accessible node of an altered wider network, and many cortical and sub-cortical regions are affected by rTMS to the DLPFC through connectivity or network interactions [128, 129]. Indirect stimulation of non-target, deeper brain structures is well established in the rTMS literature (e.g [130]), and this could mediate the therapeutic mechanisms of rTMS, but it might also lead to unintended modulation of symptoms, behaviour, or cognition in a clinically meaningful manner. For example, in a case of depression, HF-rTMS to the left-DLPFC reduced anxiety symptoms but did not change depressive symptoms [91]. However, indirect stimulation of non-target sites may also be linked to the TMS-related, new-onset psychiatric symptoms and/or diagnoses that have been reported in several studies [72, 83, 89, 114, 115]. Overall, unexpected outcomes such as these, highlight the need for better understanding of the biophysiological mechanisms of rTMS and how different parameters impact on response to rTMS in CYP with psychiatric disorders. To capture potential, unintended effects that may emerge due to indirect stimulation of non-target regions that are functionally connected to the stimulation site, broadening outcome measures will be essential in future studies. In addition, future studies should actively collect data for AEs, using a structured questionnaire (e.g [131]), in which the rater asks for each specific AE (e.g., headache).

### Limitations

Methodological heterogeneity, small sample sizes per study, and overall poor study quality, limited interpretation, and comparison across findings. It also precluded adequately powered meta-analyses of clinical, cognitive, or mood outcomes. All open-label studies and case series/studies were rated as poor quality, while RCTs had some concerns (*n* = 8) or a high (*n* = 1) risk of bias. These ratings were mainly due to a lack of detail regarding randomisation, allocation concealment and blinding. None of the randomised, sham-controlled trials assessed the integrity of blinding of raters and/or experimenters, i.e., it cannot be ruled out that rTMS-effects were due to placebo and/or bias by knowledge of group assignment.

Of note, it appears that ongoing and upcoming trials are of improved quality. Over half (65%) of the 17 trials we identified, are double-, triple-, or quadruple-blind RCTs with larger sample sizes (~ 60 on average). However, 11 of the 17 registered trials are recruiting children with ASD or MDD; thus, we cannot be sure that the same improvement in quality will be seen across other psychiatric disorders or non-registered trials.

## Conclusion

Preliminary evidence is positive but, at present, it remains insufficient to conclude that rTMS can improve clinical symptoms, mood, or cognition in CYP with a range of psychiatric disorders. This inability to make definitive statements is largely due to the methodological heterogeneity, the low quality of study designs, the small sample sizes, and the limited outcome measures, all of which limit the interpretability and comparability of findings across studies. Future studies will need to substantiate the initial evidence from existing open-label studies and small clinical trials. This will require larger, randomised, sham-controlled designs, that include longer-term follow-up periods. It will also require clinical, cognitive and mood outcomes that comprehensively capture hypothesised and also unexpected effects of rTMS. Future trials must adequately consider and address the role of the placebo response. Lastly, the studies should be aware of the protocols, measures and procedures that are in place in many comparable studies in adult populations.

## Electronic supplementary material

Below is the link to the electronic supplementary material.

Supplementary Material 1
