# Supplementary material for: Effects of repetitive transcranial magnetic stimulation in children and young people with psychiatric disorders: a systematic review
Source: Eur Child Adolesc Psychiatry. 2024 May 29;34(2):403–22. doi: 10.1007/s00787-024-02475-x (PMC11868357; doi:10.1007/s00787-024-02475-x)

*Supplementary Material S1: Data extraction*

We extracted the following information: (a) study characteristics (e.g. article title, reference, study design, sample size calculation); (b) participants (e.g. sample size, age, gender, inclusion/exclusion criteria, main disorder, illness severity, comorbidities); (c) tDCS (type of tDCS used (i.e. anodal, cathodal), site of stimulation, stimulation intensity, duration, total number of sessions); (d) comparators (sham, treatment as usual, waitlist, no comparison); (e) concurrent treatment (medication, psychotherapy, cognitive remediation); (f) outcomes (disorder-specific symptoms, mood, cognition, adverse effects).

*Supplementary Material S2: Summary of open-label studies, case series, and case studies using repetitive transcranial magnetic stimulation in children and young people with neurodevelopmental disorders*

| **Authors (year)** | **Design** | **N** | **Diagnosis** | **Mean age (range)** | **Stimulation frequency: site of stimulation** | **Stimulation protocol** | **Disorder-specific outcome measures** | **Adverse events (% of participants reported)** |
| --- | --- | --- | --- | --- | --- | --- | --- | --- |
| Ezedinma et al., (2022) | Open-label, single-arm study | 28 | ASD | 6.1 (3-9) | >9Hz, HF: DLPFC/cingulate cortex | 19 sessions; n/r; 80% RMT | CARS-R; CARS-I; CARS-ER; CARS-BU; CARS-OU; CARS-A; CARS-VR; CARS-LR; CARS-T; CARS-F; CARS-VC; CARS-NVC; CARS-A; CARS-IR; CARS-GI | Hypersalivation and “tongue-twisting” (4%)  Increased tantrums and screaming (sensory overload; 7%)  Increased emotion dysregulation (crying for no reason; 4%) |
| Assadi et al., (2020) | Open-label, single-arm study | 4 | ASD | (11-17) | 10Hz, HF: L-IPL | 11 sessions; 1000 pulses; 80% RMT | ADOS-2; SRS-2 | Reported there were ‘no AEs’ |
| Casanova et al., (2020) | Open-label, single-arm study | 19 | ASD | 14.6 (9-17) | 1Hz, LF: L- & R-DLPFC | 18 sessions; 180 pulses; 90% RMT | ABC-total; ABC-I; ABC-L; ABC-H; ABC-ST; ABC-SP; RBS-R-total; RBS-R-ST; RBS-R-SI; RBS-R-C; RBS-R-R; RBS-R-RN | Not measured or reported |
| Kang et al., (2019) | Open-label, single-arm study | 16 | ASD | 7.8 | 1Hz, LF: L- & R-DLPFC | 18 sessions; 180 pulses; 90% RMT | ABC^1^; ABC^1^-SR; ABC^1^-S; ABC^1^-BO; ABC^1^-L; ABC^1^-SA | Not measured or reported |
| Yang et al., (2019) | Open-label, single-arm study | 11 | ASD | 7.1 (3-12) | 20Hz, HF: L-IPL | 15 sessions; n/r; 50% RMT | VerBAS; ATEC-L; ATEC-S; ATEC-C; ATEC-P | Caregiver-reported transient irritability (10%) |
| Sokhadze et al., (2018) | Open-label, multi-arm study | 124 | ASD | 13.1 | 1Hz, LF: L- & R-DLPFC | 6, 12, 18 sessions; 180 pulses; 90% RMT | ABC-total; ABC-I; ABC-L; ABC-H; ABC-ST; ABC-SP; RBS-R-total; RBS-R-ST; RBS-R-SI; RBS-R-C; RBS-R-R; RBS-R-RN | Not measured or reported |
| Avirame et al., (2017) | Case study | 1 | ASD | 25 | 5Hz, HF: L- & R-DMPFC | 27 sessions; n/r; 110% RMT | IRI; AQ; YBOCS | Not measured or reported |
| Abujadi et al., (2018) | Open-label, single-arm study | 10 | ASD | (9-17) | 50Hz, iTBS: R-DLPFC | 15 sessions; n/r; 100% RMT | RBS-R; YBOCS | Reported there were ‘no AEs’ |
| Sokhadze et al., (2017) | Open-label, single-arm study | 27 | ASD | 12.5 | 0.5Hz, LF: L- & R-DLPFC | 18-sessions; 160 pulses; 90% RMT | ABC-I; ABC-L; ABC-H; ABC-ST; ABC-SP; RBS-R-total; RBS-R-ST; RBS-R-SI; RBS-R-C; RBS-R-SA; RBS-R-RN; SRS-2-A; SRS-2-CG; SRS-2-MO; SRS-2-C; SRS-2-M | Not measured or reported |
| Sokhadze et al., (2016) | Open-label, single-arm study | 23 | ASD | 13.6 | 1Hz, LF: L- & R-DLPFC | 18-sessions; 180 pulses; 90% RMT | ABC-I; ABC-L; ABC-H; ABC-ST; ABC-SP; RBS-R-total; RBS-R-ST; RBS-R-SI; RBS-R-C; RBS-R-SA; RBS-R-RN | Not measured or reported |
| Wang et al., (2016) | Open-label, single-arm study | 33 | ASD | 12.9 (7-21) | 0.5Hz, LF: L- & R-DLPFC | 18-sessions; 160 pulses; 90% RMT | ABC-I; ABC-L; ABC-H; ABC-ST; ABC-SP; RBS-R-total; RBS-R-ST; RBS-R-SI; RBS-R-C; RBS-R-SA; RBS-R-RN | Not measured or reported |
| Casanova et al., (2014) | Open-label, single-arm study | 18 | ASD | 13.1 | 0.5Hz, LF: L- & R-DLPFC | 18-sessions; 160 pulses; 90% RMT | ABC-I; ABC-L; ABC-H; ABC-ST; ABC-SP; RBS-R-total; RBS-R-ST; RBS-R-SI; RBS-R-C; RBS-R-SA; RBS-R-RN | Not measured or reported |
| Cristancho et al., (2014) | Case study | 1 | ASD | 15 | 1Hz, LF: L- & R-DLPFC | 26-sessions; 300-600 pulses; 90% RMT | Caregiver reports in mood, communication, social interaction, and behavioural regulation | Mild headaches  Jaw twitch  Transient dizziness |
| Enticott et al., (2011) | Case study | 1 | ASD | 20 | 5Hz, HF: L- and R-MPFC | 9-sessions; n/r; 100% RMT | IRI; ASQ; RAADS | Not measured or reported |
| Gómez et al., (2014) | Open-label, single-arm study | 13 | ADHD | (7-12) | 1Hz, LF: L-DLPFC | 5-sessions; 1500 pulses; 90% RMT | Parent-rated & teacher-rated SCL | Headache (70%)  Neck pain (20%)  Dizziness (10%) |
| Kahl et al., (2021) | Open-label, single-arm study | 9 | TS | 11.4 (9-15) | 1Hz, LF: SMA | 15-sessions; 1800 pulses; 100% RMT | YGTSS | Reported there were ‘no AEs’ |
| Kwon et al., (2011) | Open-label, single-arm study | 10 | TS | 9.6 (9-14) | 1Hz, LF: SMA | 10-sessions; 1200 pulses; 100% RMT | YGTSS; YTRS | Scalp pain (10%) |
| Mantovani et al., (2007) | Case study | 2 | TS | 19 (16-22) | 1Hz, LF: SMA | 10-sessions; 1200 pulses; 110% RMT | YGTSS; YBOCS | Not measured or reported |

*NOTE:* Green = Improvement; Red = No improvement; ASD = Autism Spectrum Disorder; ADHD = Attention Deficit-Hyperactivity Disorder; TS = Tourette’s Syndrome; L = Left; R = Right; iTBS = Intermittent Theta Burst Stimulation; HF = High Frequency rTMS; LF = Low Frequency rTMS; DLPFC = Dorsolateral Prefrontal Cortex; SMA = Supplementary Motor Area; DMPFC = Dorsal Medial Prefrontal Cortex; MPFC = Medial Prefrontal Cortex; IPL = Inferior Parietal Lobule; RMT = Resting Motor Threshold; CARS = Childhood Autism Rating Scale; CARS-R = Relating to People; CARS-I = Imitation; CARS-ER = Emotional Response; CARS-BU = Body Use; CARS-OU = Object Use; CARS-A = Adaptation to Change; CARS-VR = Visual Response; CARS-LR = Listening Response; CARS-T = Taste/Smell/Touch Response and Use; CARS-F = Fear or Nervousness; CARS-VC = Verbal Communication; CARS-NVC = Non-Verbal Communication; CARS-A = Activity Level; CARS-IR = Level of Consistency of Intellectual Response; CARS-GI = General Impression; RBQ = Repetitive Behaviours Questionnaire-2; SSP = Short Sensory Profile; SDQ = Strengths and Difficulties Questionnaire; ABC = Aberrant Behaviour Checklist; ABC-I = Aberrant Behaviour Checklist – Irritability; ABC-L = Aberrant Behaviour Checklist – Lethargy; ABC-H = Aberrant Behaviour Checklist – Hyperactivity; ABC-ST = Aberrant Behaviour Checklist – Stereotypy; ABC-SP = Aberrant Behaviour Checklist - Speech; RBS-R = Repetitive Behaviour Scale – Revised; RBS-R-ST = Repetitive Behaviour Scale – Revised – Stereotypy; RBS-R-SI Repetitive Behaviour Scale – Revised – Self-Injury; RBS-R-C = Repetitive Behaviour Scale – Revised – Compulsivity; RBS-R-R = Repetitive Behaviour Scale – Revised – Rituals; RBS-R-RN = Repetitive Behaviour Scale – Revised – Restriction; ABC^1^ = Autism Behaviour Checklist; ABC^1^-SR = ABC^1^-S = Autism Behaviour Checklist - Sensory; ABC^1^-BO = Autism Behaviour Checklist – Body & Object; ABC^1^-L = Autism Behaviour Checklist – Language; ABC^1^-SA = Autism Behaviour Checklist – Social & Adaptive; verBAS = Verbal Behaviour Assessment Scale; ATEC = Autism Treatment Evaluation Checklist; ATEC-S = Autism Treatment Evaluation Checklist – Speech/Language Communication; IRI = Interpersonal Reactivity Index; AQ = Autism Quotient; YBOCS = Yale-Brown Obsessive Compulsive Scale; RAADS = The Ritvo Autism Asperger Diagnostic Scale; SCL = Symptom Checklist; YGTSS = Yale Global Tic Severity Scale; YTRS = Yale Tic Rating Scale; AE = Adverse Event.

*Supplementary Material S3: Summary of open-label studies, case series, and case studies using repetitive transcranial magnetic stimulation in children and young people with psychiatric disorders*

| **Authors (year)** | **Design** | **N** | **Diagnosis** | **Mean age (range)** | **Stimulation frequency: site of stimulation** | **Stimulation protocol** | **Disorder-specific outcome measures** | **Adverse events (% of participants reported)** |
| --- | --- | --- | --- | --- | --- | --- | --- | --- |
| Gordon et al., (2021) | Open-label, two-arm study | 14 | MDD | 16.5 (14-18) | 10Hz, HF: L-DLPFC  1Hz, LF: R-DLPFC | 20 sessions; 1800 pulses; 120% RMT | CDRS-R; MFQ-AR; MFQ-PR; CGI-I; CGI-S | Lethargy/drowsiness (100%)  Headache (82%) |
| Shere et al., (2021) | Open-label, single-arm study | 26 | MDD | 15.8 (12-18) | 50Hz, iTBS: L-DLPFC  50Hz, cTBS: R-DLPFC | 10 sessions; 3600 pulses; 80% RMT | CDRS-R | 53.8% reported ≥1 AE:  Headache (35%)  Neck pain (15%)  Scalp irritation (12%)  Affective switch (8%) Lachrymation (4%) |
| Zhang et al., (2021) | Open-label, single-arm study | 29 | MDD | (10-17) | 10Hz, HF: L-DLPFC  1Hz, LF: R-DLPFC | 10 sessions; 2400 pulses; 120% RMT | HAM-D; HAMD-SI | Headaches (7%)  Muscoskeletal discomfort (14%) |
| Kallel & Brunelin (2020) | Case study | 1 | MDD | 18 | 20Hz, HF: L-DLPFC  1Hz, LF: R-DLPFC | 30 sessions; 360 pulses; 120% RMT | MADRS | Seizure occurred during the third session of day-2 (5-session per day, 4 consecutive days: 20 total) and resolved spontaneously. 12-days after seizure, rTMS restarted with no AEs reported |
| Sonmez et al., (2020) | Open-label, single-arm study | 21 | MDD | 15.9 (13-19) | 10Hz, HF: L-DLPFC | 30 sessions; 3000 pulses; 120% RMT | CDRS-R; QIDS-A_17_-SR-I; QIDS-A_17_-SR-H | Withdrew due to tolerability issues (14%)  Emergent suicidal ideation (5%) |
| Zhang et al., (2020) | Open-label, single-arm study | 42 | MDD | 15.2 (10-18) | 10Hz, HF: L-DLPFC | 10-20 sessions; 2400 pulses; 80-120% RMT | HAM-A; HAM-D | Dizziness (2%) |
| Zhang et al., (2019) | Open-label, single-arm study | 42 | MDD | 14.6 (10-18) | 10Hz, HF: L-DLPFC | 10-20 sessions; 2400 pulses; 120% RMT | HAM-D | Transient headaches (7%)  Musculoskeletal pain (5%) |
| Yanbin et al., (2019) | Case study | 1 | MDD | 15 | 1Hz, LF: R-DLPFC | 6 sessions; 1200 pulses; 120% RMT | - | Visual hallucinations after session-6; diagnosed with rTMS-induced Charles Bonnet Syndrome; resolved 7-days after rTMS terminated |
| MacMaster et al., (2019) | Open-label, single-arm study | 32 | MDD | 17.6 (12-22) | 10Hz, HF: L-DLPFC | 15 sessions; 3000 pulses; 120% RMT | HAM-D; CDRS; BDI | *Headaches (31%)  Neck pain (22%)  Unpleasant tingling (19%)  Nausea (9%)  Light headedness (13%) |
| Rosenich et al., (2019) | Open-label, single-arm study | 15 | MDD | 20.7 (17-25) | 10Hz, HF: L-DLPFC  1Hz, LF: R-DLPFC | 18 sessions; 2400 pulses; 110% RMT | HAM-D; MADRS; SDS | Measured, not reported |
| Dhami et al., (2019) | Open-label, single-arm study | 20 | MDD | 20.9 (16-24) | 50Hz, iTBS: L-DLPFC  50Hz, cTBS: R-DLPFC | 10 sessions; 1800 pulses; 80% AMT | HAM-D; CDRS-R; BDI | *Headache (70%) |
| Croarkin et al., (2018) | Open-label, single-arm study | 19 | MDD | 16 (13-19) | 10Hz, HF: L-DLPFC | 30 sessions;  3000 pulses; 120% RMT | CDRS-R | Not measured or reported |
| Pan et al., (2018) | Case series | 3 | MDD | 16 (15-17) | 10Hz, HF: L-DLPFC | 7 sessions; 6000 pulses; 100% RMT | BSI-CV; MADRS | Two observed hypomanic symptoms, one of which was diagnosed with hypomania |
| Wall et al., (2016) | Open-label, single-arm study | 10 | MDD | 15.9 (13-17) | 10Hz, HF: L-DLPFC | 30 sessions; 3000 pulses; 120% RMT | CDRS-R, QIDS-A17-SR | One withdrawal due to tolerability issues; one hospitalised due to worsening depression symptoms; suicidality worsened in two patients; most common AE: transient scalp discomfort |
| Cullen et al., (2016) | Case study | 1 | MDD | 23 | 18Hz, HF: n/r | 8 sessions; 1980 pulses; 120% RMT | CDRS-R | During 8^th^ session, the patient had a generalised, tonic-clonic seizure that resolved spontaneously. |
| Best & Griffin (2015) | Case study | 1 | MDD | 23 | 1Hz, LF: ACC | 21 sessions; n/r; 115% RMT | BDI; PAI | Not measured or reported |
| Nakama et al., (2014) | Case study | 1 | MDD & PTSD | 24 | 10Hz, HF: L-DLPFC | 22 sessions; 3000 pulses; 120% RMT | PCL-M; BDI | Not measured or reported |
| Yang et al., (2014) | Open-label, single-arm study | 6 | MDD | 18.7 (12-22) | 10Hz, HF: L-DLPFC | 15 sessions; 3000 pulses; 120% RMT | HAM-D; BDI | *”Some patients reported minor adverse effects of scalp discomfort or headaches during rTMS administration” |
| Segev et al., (2014) | Case study | 1 | MDD | 17 | 10Hz, HF: L-DLPFC | 20 sessions; 1680 pulses; 100% RMT | BDI; CDRS-R | Not measured or reported |
| Chiramberro et al., (2013) | Case study | 1 | MDD | 16 | 10Hz, HF: L-DLPFC | 12 sessions; 3000 pulses; n/r | n/r | TMS-related seizure on 12^th^ day of stimulation, complicated by alcohol |
| Wall et al., (2013) | Open-label, single-arm study | 18 | MDD | 16.3 (13-17) | 10Hz, HF: L-DLPFC | 30 sessions; 3000 pulses; 120% RMT | n/r | Not measured or reported |
| Mayer et al., (2012) | Open-label, single-arm study | 8 | MDD | 20.4 (19-22) | 10Hz, HF: L-DLPFC | 14 sessions; n/r; 80% RMT | BDI; CDRS-R | Not measured or reported |
| Hu et al., (2011) | Case study | 1 | MDD | 15 | 10Hz, HF: L-DLPFC | 1 session; n/r; 80% RMT | n/r | TMS-related seizure during first session with 8-hour hypomanic symptom sequela |
| Bloch et al., (2008) | Open-label, single-arm study | 9 | MDD | 17.3 (16-18) | 10Hz, HF: L-DLPFC | 14 sessions; n/r; 80% RMT | n/t | Headache (55%) |
| Loo et al., (2006) | Case study | 2 | MDD | 16 | 10Hz, HF: L-DLPFC | 29 sessions; n/r; 80% RMT | MADRS; BDI; CES-DC | Not measured or reported |
| Garg et al., (2019) | Case study | 1 | SCZ | 18 | 20Hz, HF: L-DLPFC | 20-sessions; 2000 pulses; 100% RMT | PANSS; SANS; YBOCS | New onset OCD diagnosis following rTMS |
| Purushotham et al., (2018) | Case study | 1 | SCZ | 15 | 50Hz, iTBS: L-motor cortex | n/r; 600 pulses; 80% AMT | n/r | TBS-related seizure within 30-seconds of first session |
| Blanco-Lopez et al., (2016) | Case study | 1 | SCZ | 18 | 1Hz, LF: L-TPP | 20-sessions; 1200 pulses; 90% RMT | PHQ-9 | Not measured or reported |
| Giesel et al., (2012) | Case study | 1 | SCZ | 22 | 1Hz, LF: L-STG | 20-sessions; 100-200 pulses; 80-100% RMT | AHRS | Not measured or reported |
| Jardri et al., (2012) | Case series | 10 | SCZ | 15.5 | 1Hz, LF: L-TPP | 10-sessions; 1200 pulses; 90% RMT | AHRS | Minor discomfort reported |
| Jardri et al., (2009) | Case study | 1 | SCZ | 11 | 1Hz, LF: L-TPJ | 10-sessions; 1000 pulses; 100% RMT | AHRS | Reported there were ‘no AES’ |
| Fitzgerald et al., (2006) | Case study | 1 | SCZ | 18 | 1Hz, LF: L-TPP | 10-sessions; n/r; 90% RMT | PANSS; HCS | Not measured or reported |
| Mikellides et al., (2021) | Case study | 1 | OCD | 19 | 50Hz, iTBS: L-DLPFC  50Hz, cTBS: R-DLPFC | 50-sessions; 200 pulses; 120% RMT | - | Seizure lasting 2-3 minutes occurred during session 7. rTMS restarted 8-days later. 10-days after final session, a new seizure episode occurred which resolved without medication or hospitalisation |
| Mehta et al., (2020) | Case study | 1 | OCD | 21 | 50Hz, iTBS: pre-SMA | 27-sessions; 600 pulses; 90% RMT | YBOCS | Not measured or reported |
| Verma, Kumar & Kuppili (2018) | Case study | 1 | OCD | 23 | 1Hz, LF: SMA | 35-sessions; 1200 pulses; 85% RMT | YBOCS | Not measured or reported |
| Deftereos & Georgonikou (2017) | Case study | 1 | OCD | 19 | 1Hz, LF: SMA | 20-sessions; 600 pulses; 100% RMT | YBOCS | Not measured or reported |
| Holbert & Witter (2017) | Case study | 1 | OCD | 18 | 1Hz, LF: SMA | 30-sessions; 1800 pulses; n/r | YBOCS | Not measured or reported |
| Choudhary et al., (2017) | Case study | 1 | AN | 23 | 10Hz, HF: L-DLPFC | 21 sessions; 1000 pulses; 110% RMT | BMI | Not measured or reported |
| Baczynski et al., (2014) | Case study | 1 | BED | 19 | 10Hz, HF: L-DLPFC | 20-sessions; 2400 pulses; 120% RMT | BMI; BES; no. of binge eating episodes p/w | Not measured or reported |
| McClelland et al., (2013) | Case study | 1 | AN | 23 | 10Hz, HF: L-DLPFC | 10-sessions; 1000 pulses; 110% RMT | BMI; EDE-Q | Not measured or reported |
| Sharma et al., (2018) | Case study | 1 | Catatonia | 16 | 10Hz, HF: L-DLPFC | 19-sessions; 1200 pulses; 100% RMT | BFCRS | Not measured or reported |
| Saba et al., (2002) | Case study | 1 | Catatonia | 18 | 10Hz, HF: L-DLPFC | 10-sessions; 1600 pulses; 80% RMT | BFCRS | Not measured or reported |
| Svěrák et al., (2019) | Case series | 3 | BPD | 20.3 (18-23) | 10Hz, HF: R-DLPFC | 15-sessions; 1500 pulses; 110% RMT | CGI | Not measured or reported |
| Arbabi et al., (2013) | Case study | 1 | BPD | 22 | 10Hz, HF: L-DLPFC | 10-sessions; n/r; 100% RMT | BIS-11; BPDSI | Not measured or reported |
| Paes et al., (2013) | Case study | 1 | SAD | 23 | 1Hz, LF: R-vmPFC | 12-sessions; 1500 pulses; 120% RMT | LSAS | Not measured or reported |
| Cuppone et al., (2021) | Case study | 1 | IGD | 21 | 10Hz, HF: L-DLPFC | 26-sessions; 2400 pulses; 100% RMT | IGDS9-SF; IAT; VAS current craving | Not measured or reported |

*NOTE:* *AEs assessed actively, i.e., using a questionnaire. All other studies assessed AEs passively – i.e., via spontaneously reported feedback; Green = improvement; Red = no improvement; MDD = Major Depressive Disorder; PTSD = Post-Traumatic Stress Disorder; SCZ = Schizophrenia; OCD = Obsessive Compulsive Disorder; AN = Anorexia Nervosa; BED = Binge Eating Disorder; BPD = Borderline Personality Disorder; SAD = Social Anxiety Disorder; IGD = Internet Gaming Disorder; L = Left; R = Right; iTBS = Intermittent Theta Burst Stimulation; cTBS = Continuous Theta Burst Stimulation; HF = High Frequency rTMS; LF = Low Frequency rTMS; DLPFC = Dorsolateral Prefrontal Cortex; TPJ = Temporoparietal Junction; TPP = Temporoparietal Cortex; ACC = Anterior Cingulate Cortex; vmPFC = Ventromedial Prefrontal Cortex; SMA = Supplementary Motor Area; STG = Superior Temporal Gyrus; MPFC = Medial Prefrontal Cortex; IPL = Inferior Parietal Lobule; RMT = Resting Motor Threshold; CDRS-R = Children’s Depression Rating Scale-Revised; MFQ = Moods and Feelings Questionnaire; C-SSRS = Columbia-Suicide Severity Rating Scale; HAM-D = Hamilton Depression Rating Scale; HAM-D-SI = Hamilton Depression Rating Scale – Suicidal Ideation Item; MADRS = Montgomery-Åsberg Depression Rating Scale; QIDS-A^17^-SR = The Quick Inventory of Depressive Symptomatology Adolescent Version; QIDS-A^17^-SR-I = The Quick Inventory of Depressive Symptomatology Adolescent Version – Insomnia Item; QIDS-A^17^-SR-H = The Quick Inventory of Depressive Symptomatology Adolescent Version – Hypersomnia Item; BDI = Beck Depression Inventory; SDS = Zung Self-Rating Depression Scale; BSI-CV = Beck Scale for Suicidal Ideation – Chinese Version; PCL = Posttraumatic Stress Disorder Checklist; CES-DC = Center for Epidemiological Studies Depression Scale for Children; PANSS = Positive and Negative Syndrome Scale; SANS = Scale for the Assessment of Negative Symptoms; PHQ-9 = Patient Health Questionnaire – 9; AHRS = Auditory Hallucination Rating Scale; HCS = Hallucination Change Scale; SCL = Symptom Checklist; YGTSS = Yale Global Tic Severity Scale; YTRS = Yale Tic Rating Scale; BMI = Body Mass Index; BES = Binge Eating Scale; EDE-Q = Eating Disorder Examination Questionnaire; BFCRS = Bush Francis Catatonia Rating Scale; CGI = Clinical Global Impression; BIS = Barratt Impulsiveness Scale – Version 11; BPDSI = The Borderline Personality Severity Index; LSAS = Liebowitz Social Anxiety Scale; IGDS9-SF = Internet Gaming Disorder Scale – Short-Form; IAT = Internet Addiction Test; VAS = Visual Analogue Scale.

*Supplementary Material S4: Summary of ongoing trials using repetitive transcranial magnetic stimulation in children and young people with psychiatric disorders*

| **Trial ID (Status)** | **Design** | **N^(a)^** | **Diagnosis** | **Age range** | **Control** | **Stimulation frequency: site of stimulation** | **Protocol** | **POM** |
| --- | --- | --- | --- | --- | --- | --- | --- | --- |
| NCT05927792 (Not yet recruiting) | Double-blind, sham-controlled RCT | 200 | ASD | 4-10 | Sham rTMS | 50Hz, cTBS: L-M1 | 50-sessions; 18000 pulses; 80% RMT | SRS; CCDI; PPVT; MAIN; CGI; VABS; EEG |
| NCT05472870 (Enrolling by invitation) | Open-label, single-arm study | 30 | ASD | 4-16 | None | n/r, cTBS: L-M1 | 5-sessions; n/r; 80% RMT | SRS; CARS; BRIEF; Conners; RBS-R; PPVT; CCDI; CGI |
| NCT05238298  (Completed^(a)^) | Open-label, single-arm study | 20 | ASD | 6-10 | None | n/r, cTBS: L-M1 | 10-sessions; n/r; n/r | SRS; RBS-R; ADHD-IV; BRIEF; CARS; CGI; CANTAB; CPT; EEG; fNIRS |
| NCT04972136 (Recruiting) | Double-blind, sham-controlled RCT | 80 | ASD | 16-29 | Sham rTMS | 50Hz, iTBS: L-DLPFC  50Hz, cTBS: R-DLPFC | 30-sessions; 600 pulses; 90% RMT | HAM-D; BSI |
| NCT05235919 (Recruiting) | Triple-blind, sham-controlled RCT | 50 | ASD | 9-18 | Sham rTMS | n/r | 15-sessions; n/r; n/r | EDIRD; RSQ-FS; ABC-I; ERC; CGI; Go/No-Go Task; DTI; fMRI |
| NCT04532190 (Not yet recruiting) | Double-blind, sham-controlled RCT | 30 | ADHD | 8-16 | Sham rTMS | 10Hz, HF: R-SFG | n/r; 3000 pulses; 120% RMT | Conners-3 |
| NCT05544071 (Not yet recruiting) | Triple-blind, sham-controlled RCT | 82 | MDD | 13-18 | Sham rTMS | 10Hz, HF: L-DLPFC | n/r; n/r; n/r | SHAPS; HAM-D; MADRS; CV-TEPS; BSI; ISI; CGI; fMRI; THINC-it® |
| NCT05465928 (Recruiting) | Triple-blind, sham-controlled RCT | 120 | MDD | 13-25 | Sham rTMS | 1Hz or 10Hz, LF or HF: DMPFC or OCC | 20-sessions;^(f)^; n/r | HAM-D; RBANS; CGI-S; MADRS; ALFF |
| NCT02611206  (Completed^(b)^) | Open-label, single-arm study | 24 | MDD | 12-18 | None | 10Hz, HF: n/r | 30-sessions; n/r; 80% RMT | CDRS-R; BDI; C-SSRS; TEPS; SHAPS; IDAS; YMRS |
| NCT03708172  (Completed^(c)^) | Quadruple-blind, sham-controlled trial | 30 | MDD | 16-24 | Sham-rTMS + CT | n/r, iTBS: L-DLPFC  n/r, cTBS: R-DLPFC | 20-sessions; n/r; n/r | HAM-D; BDI; CDRS-R |
| NCT03363919 (Completed^(d)^) | Double-blind, active-comparator RCT | 120 | MDD | 12-18 | 1Hz vs. 10Hz | 10Hz, HF: L-DLPFC  1Hz, LF: L-DLPFC | 36-sessions; 2400 pulses; 120% RMT | CDRS-R |
| NCT03986658  (Not yet recruiting) | Open-label, single-arm study | 19 | MDD | 15-17 | None | n/r: n/r | 1-10 sessions; 30000 pulses^(e)^; n/r | CDI-2; CDRS-R; C-GAS; MMSE; HLVT-R |
| NCT05249140 (Not yet recruiting) | Double-blind, sham-controlled RCT | 24 | AN | 12-18 | Sham rTMS | 1Hz, LF: R-DLPFC | 20 sessions; 1800 pulses; 110% RMT | MASC-2; BMI |
| NCT04846517 (Not yet recruiting) | Open-label, single-arm study | 45 | AN | 14-24 | None | 50Hz, iTBS: L-DLPFC  1Hz, LF: SMA | n/r; 1800 pulses; n/r | YBC-EDS |
| NCT03844919 (Recruiting) | Double-blind, sham-controlled RCT | 50 | TS | 6-18 | Sham-rTMS + CBIT | 1Hz, LF: SMA | 20 sessions; 1800 pulses; 100% RMT | YGTSS; SMA-GABA; |
| NCT03628703 (Recruiting) | Open-label, single-arm study | 15 | TS | 10-17 | None | n/r, iTBS; pre-SMA | n/r; n/r; n/r | SSRT; SMA-GABA; YGTSS |
| NCT04578912 (Recruiting) | Triple-blind, multi-arm, sham-controlled RCT | 60 | Tic Disorders | 12-21 | Sham-rTMS + CBIT | 1Hz, LF: SMA  n/r, cTBS: SMA | 10 sessions; n/r; n/r | fMRI; TST |

*Note:* n/r = not reported; RCT = Randomised Controlled Trial; ASD = Autism Spectrum Disorder; MDD = Depression; AN = Anorexia Nervosa; TS = Tourette’s Syndrome; rTMS = repetitive transcranial magnetic stimulation; CT = Cognitive Training; CBIT = Comprehensive Behavioural Intervention for Tics; L = Left; R = Right; LF = Low-Frequency; HF = High-Frequency; cTBS = continuous theta burst stimulation; iTBS = intermittent theta burst stimulation; M1 = Primary Motor Cortex; DLPFC = Dorsolateral Prefrontal Cortex; SMA = Supplementary Motor Area; SFG = Superior Frontal Gyrus; DMPFC = Dorsomedial Prefrontal Cortex; OCC = Occipital Cortex; RMT = Resting Motor Threshold; SRS = Social Responsiveness Scale; CCDI = Chinese Communicative Development Inventory; MAIN = Multilingual Assessment Instrument for Narratives; VABS = Vineland Adaptive Behaviour Scales; RBS-R = Repetitive Behaviour Scale-Revised; PPVT = Peabody Picture Vocabulary Test; EEG = Electroencephalography; BRIEF = Behaviour Rating Inventory of Executive Function; ADHD-IV = ADHD Rating Scale-IV; BRIEF = Behaviour Rating Inventory of Executive Function; CARS = Childhood Autism Rating Scale; CGI = Clinical Global Impression; CANTAB = Cambridge Neuropsychological Test Automated Battery; CPT = Continuous Performance Tests; EEG = Electroencephalogram; fNIRS = Functional Near-Infrared Spectroscopy; HAM-D = Hamilton Depression Rating Scale; BSI = Beck Scale for Suicide Ideation; RBANS = Repeatable Battery for the Assessment of Neuropsychological Status; ALFF = Amplitude of Low-Frequency Fluctuation; CV-TEPS = Chinese Version of Temporal Experience of Pleasure Scale; ISI = Insomnia Severity Index; SHAPS = Snaith Hamilton Pleasure Scale; EDIRD = Emotion Dysregulation Index Reactivity and Dysphoria; RSQ-FS = Response to Stress Questionnaire – Family Stress; ABC = Aberrant Behaviour Checklist – Irritability Subscale; ERC = Emotion Regulation Checklist; DTI = Diffusion Tensor Imaging; CDRS-R = Child Depression Rating Scale-Revised; BDI = Beck Depression Inventory; C-SSRS = Columbia Suicide Severity Rating Scale; TEPS = Temporal Experience of Pleasure Scale; SHAPS = Snaith-Hamilton Pleasure Scale; IDAS = Depression and Anxiety Symptoms; YMRS = Young Mania Rating Scale; CDI-2 = Child Depression Inventory 2; C-GAS = Children’s Global Assessment Scale; MMSE = Mini-Mental State Examination; HLVT-R = Hopkins Verbal Learning Test-Revised; MASC-2 = Multidimensional Anxiety Scale for Children; BMI = Body Mass Index; YBC-EDS = Yale-Brown-Cornell Eating Disorder Scale; YGTSS = Yale Global Tic Severity Scale; SMA-GABA = Gamma-aminobutyric acid in the SMA; SSRT = Stop Signal Reaction Time; TST = Tic Suppression Task.

^(a)^ April 2022

^(b)^ December 2021

^(c)^ May 2019

^(d)^ March 2023

^(e)^ All participants receive a maximum of 30,000 pulses over the treatment course

^(f)^ Number of pulses will be based on neuroimaging biomarkers extracted via machine learning method

*Supplementary Material S4: Results of risk of bias assessment*


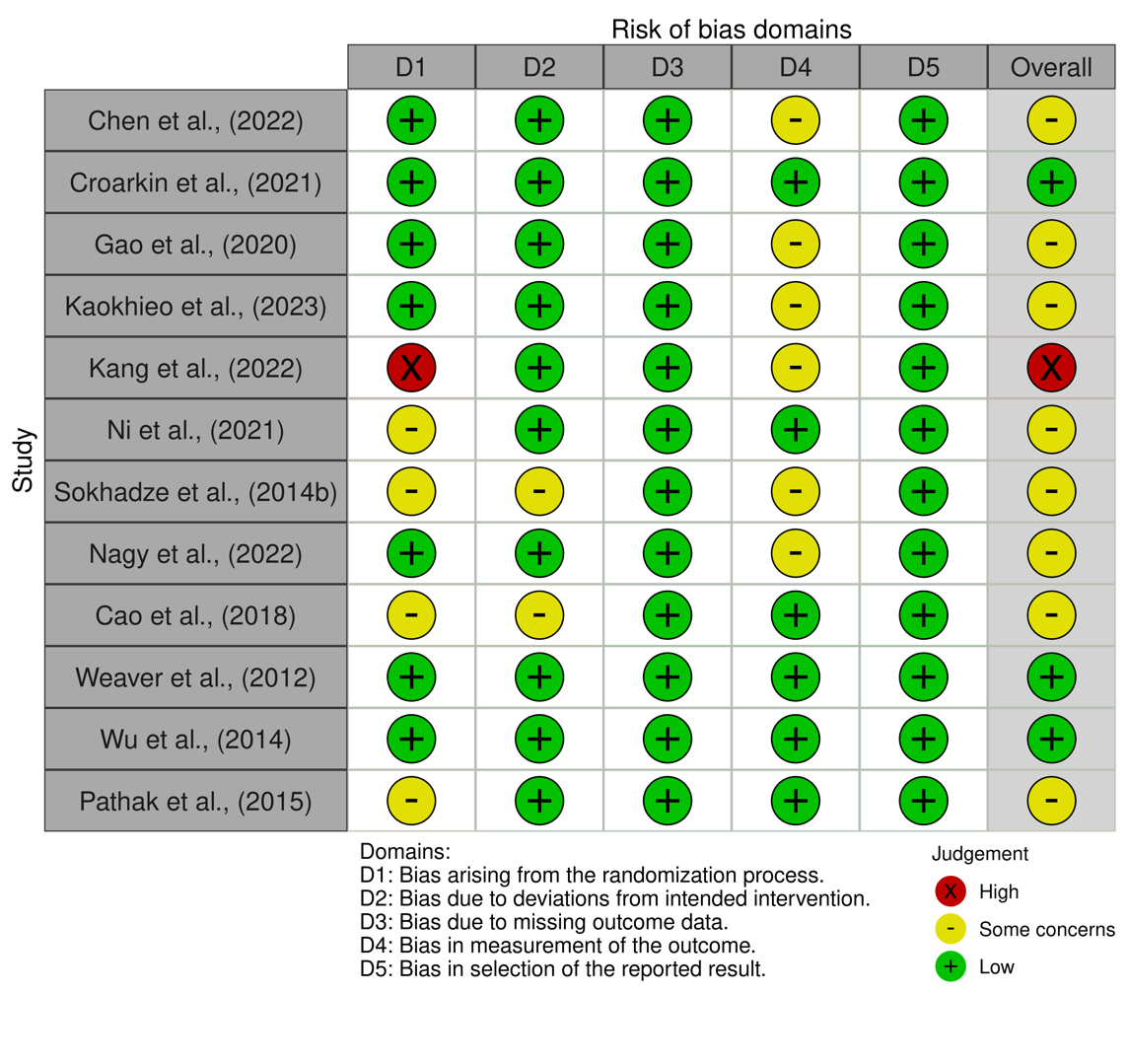


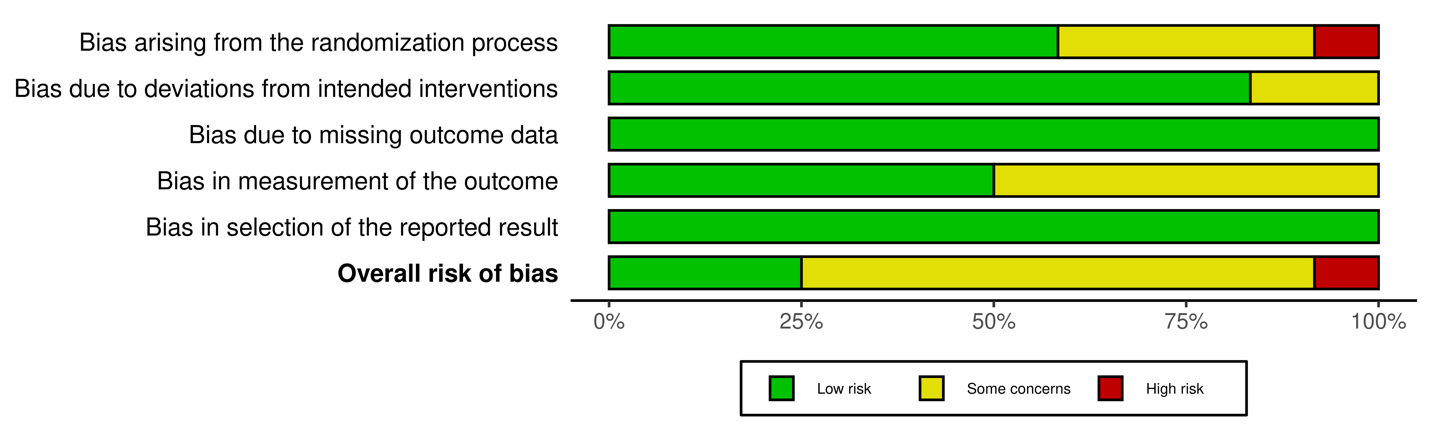

Supplement: Supplementary file 1 — Supplementary Material 1 [file 787_2024_2475_MOESM1_ESM.docx]
